# Comparative Mitogenomic and Phylogenetic Insights of Three *Aulacophora* Species (Coleoptera: Chrysomelidae) Associated with Cucurbit Crops

**DOI:** 10.3390/life16081231

**Published:** 2026-07-25

**Authors:** Liancheng Liu, Huanhuan Li, Gonghua Lin, Tianjuan Su, Fang Zhao, Bo He, Zuhao Huang

**Affiliations:** Key Laboratory of Jiangxi Province for Biological Invasion and Biosecurity, School of Life Sciences, Jinggangshan University, Ji’an 343009, China; liuliancheng1212@163.com (L.L.); hhli1105@163.com (H.L.); lingong-hua@jgsu.edu.cn (G.L.); sutianjuan126@126.com (T.S.); zf_lgh@163.com (F.Z.)

**Keywords:** *Aulacophora*, mitochondrial genome, comparative genomics, genetic diversity, phylogeny

## Abstract

This study sequenced and comparatively analyzed the mitochondrial genomes of three agriculturally important *Aulacophora* pests: the polyphagous *A. indica*, the oligophagous *A. lewisii*, and the early-diverging *A. nigripennis*. All three mitogenomes contained the typical 37 genes with conserved gene order. However, total length varied substantially (15,258–18,766 bp), driven primarily by control region length variation. All species exhibited pronounced AT bias, and relative synonymous codon usage analysis revealed marked interspecific divergence. Phylogenetic analysis based on 13 protein-coding genes resolved *A. nigripennis* as the basal lineage and *A. lewisii* and *A. indica* as closely related sister species. Both widespread species displayed maximum haplotype diversity (*Hd* = 1.000); however, *A. indica* exhibited approximately 2.4-fold higher nucleotide diversity (*Pi* = 0.00478) than *A. lewisii* (*Pi* = 0.00198), indicating differential population genetic architectures between the two species. These findings established a comparative mitogenomic framework for *Aulacophora* and provide baseline data for future phylogenomic and phylogeographic investigations.

## 1. Introduction

The mitochondrial genome (mitogenome) of most metazoan is a double-stranded circular DNA molecule that contains 13 protein-coding genes, 22 tRNA genes, two rRNA genes, and a large non-coding A + T-rich region (also referred to as the control region) [1]. Due to its small genome size, maternal inheritance, conserved gene arrangement, and high evolutionary rate [2], the mitogenome has been widely used for species identification, phylogenetic inference, population structure analysis, and phylogeographic studies [2,3].

The genus *Aulacophora* (Coleoptera: Chrysomelidae) comprises a group of phytophagous pests intimately associated with plants of the family Cucurbitaceae. Tissues of cucurbit species typically contain cucurbitacins—tetracyclic triterpenoids that are broadly toxic to most metazoans [4]. Intriguingly, however, these same compounds act as potent feeding stimulants for *Aulacophora* beetles, a trait that has driven the specialization of this lineage as obligate herbivores of cucurbit hosts [5,6]. Both the adult and larval stages inflict significant damage: adults defoliate plants and scarify tender stems, flowers, and developing fruits, whereas larvae attack the root system—often inducing progressive wilting and subsequent plant mortality [7,8]. Within this genus, species such as *A. foveicollis* and *A. indica* are recognized as the most prevalent and destructive polyphagous pests, affecting a broad spectrum of economically important cucurbit crops, including melon, cucumber, pumpkin, bottle gourd, wax gourd, watermelon, and muskmelon [9,10,11]. In marked contrast, *A. lewisii* exhibits a largely oligophagous feeding habit, with a strong preference for loofah (*Luffa* spp.) [12]. Members of this genus are prone to eruptive population outbreaks, demonstrate considerable ecological plasticity, and present formidable management challenges, thereby constituting a persistent constraint to the sustainable intensification of cucurbit production systems [8,13,14].

Although several studies have documented the biological characteristics of *Aulacophora* beetles on various cucurbit crops [15], species identification within this genus has historically relied on morphological criteria. Early classifications grouped species according to elytral coloration [16]; however, such color patterns are now recognized as variable within species and thus unreliable as sole diagnostic characters. Modern taxonomic revisions have established that the morphology of male genitalia is constant within species and, in most cases, largely different between species, thereby providing reliable diagnostic criteria for species delimitation [14]. Nevertheless, molecular data—including mitochondrial genomes—offer an independent and complementary line of evidence for species identification. They are particularly valuable for identifying immature stages, fragmentary specimens, and cryptic species complexes where morphological diagnosis may be inconclusive [17,18]. Moreover, molecular markers are indispensable for inferring population genetic structure and evolutionary history, which cannot be fully resolved through morphological approaches alone [19]. Although DNA barcoding approaches can facilitate the taxonomic placement of these pest species [20], comprehensive analyses that elucidate deeper evolutionary relationships within the genus remain underexplored. Consequently, additional molecular data and robust phylogenetic investigations are still lacking [21,22,23,24]. In light of the considerable economic impact of this genus, a detailed examination of the evolutionary relationships and population genetic architecture of its major pest species is urgently warranted.

In the present study, we selected three *Aulacophora* species based on two explicit criteria: agricultural importance and feeding strategy. *A. indica* is the most widely reported polyphagous pest within the genus, inflicting damage on a broad spectrum of economically important cucurbit crops, including melon, cucumber, pumpkin, watermelon, and muskmelon [9,10,11]. *A. lewisii* represents a predominantly oligophagous species with a strong preference for loofah (*Luffa* spp.) [12], thereby constituting a natural ecological contrast to the polyphagous *A. indica*. *A. nigripennis* has been inferred as an early-diverging lineage within the genus based on both morphological and molecular evidence [20], providing a critical root for phylogenetic analyses. We sequenced, assembled, and annotated the mitogenomes of these three *Aulacophora* species. We then systematically compared their mitochondrial genomic characteristics and constructed a phylogenetic tree to elucidate the evolutionary relationships among them. Furthermore, we employed population genetic analyses to assess genetic diversity and population structure. This study aimed to (1) characterize and compare the mitogenomic architecture of these three agriculturally important *Aulacophora* species, with a focus on genome size variation, control region dynamics, and codon usage patterns; (2) reconstruct a robust phylogenetic framework for these taxa; and (3) examine the potential association between ecological traits (host-plant breadth) and genetic diversity. As a foundational study with a limited taxonomic sample, this work established a comparative mitogenomic framework for the genus, providing a baseline that will guide future phylogenomic and phylogeographic investigations encompassing a broader diversity of *Aulacophora* species across their geographic ranges. The findings from this study will provide a theoretical foundation for exploring the ecological adaptability of these leaf beetles and for developing integrated pest management strategies.

## 2. Materials and Methods

### 2.1. Sample Collection and Species Identification

In 2019 and 2023, a total of 11 adult specimens representing three *Aulacophora* species (*A. indica*, *A. lewisii*, and *A. nigripennis*) were selected from collections across China (Table 1). All specimens were preserved in absolute ethanol and stored at −20 °C. All specimens were examined under a stereomicroscope. For *A. indica* and *A. nigripennis*, morphological identification was performed primarily using the dichotomous keys and descriptions of male genitalia provided by [11,14]. For *A. lewisii*, which is not included in [11,14], identification was based on the detailed morphological descriptions and distribution records presented in [25] and other relevant Chinese faunal works, with male genitalia serving as the primary diagnostic criterion. Furthermore, as an independent verification, the *cox1* gene sequences obtained from each specimen were compared against the NCBI nucleotide database (BLASTn). Total genomic DNA was extracted from the thoracic tissue of each individual using the QIAGEN DNeasy Blood and Tissue Kit (QIAGEN, Hilden, Germany), following the manufacturer’s instructions.

**Table 1:** Collection information for three *Aulacophora* species.

Species	Sample ID	Location	Collection Date	Longitude (E)	Latitude (N)	Altitude (m)	Accession No.
*A. indica*	Aind_HT03	Hantai, Shanxi	10 August 2023	107.04	33.12	536	PZ334452
	Aind_HX02	Huaxi, Guizhou	6 August 2023	106.66	26.52	1105	PZ334453
	Aind_LC02	Longchuan, Guangxi	31 July 2023	106.96	22.47	194	PZ334454
	Aind_ML03	Mengla, Yunnan	2 August 2023	101.26	21.93	553	PZ334455
	Aind_XZ04	Xuzhou, Sichuan	6 August 2023	104.59	28.73	292	PZ334456
	-	Zhijiang, Hubei	6 May 2019	111.83	30.52	-	NC_047467
*A. lewisii*	Alew_HT02	Hantai, Shanxi	10 August 2023	107.04	33.12	536	PZ334457
	Alew_HX01	Huaxi, Guizhou	6 August 2023	106.66	26.52	1105	PZ334458
	Alew_LC01	Longchuan, Guangxi	31 July 2023	106.96	22.47	194	PZ334459
	Alew_ML01	Mengla, Yunnan	2 August 2023	101.26	21.93	553	PZ334460
	Alew_XZ01	Xuzhou, Sichuan	6 August 2023	104.59	28.73	292	PZ334461
	-	-	-	-	-	-	NC_039712
*A. nigripennis*	Anig_HZH02	Wuyi, Zhejiang	1 November 2019	119.86	28.86	122	PZ358535

Note: Two sequences (GenBank accession numbers: NC_047467 and NC_039712) were downloaded from GenBank; the remaining 11 were newly generated, totaling 13 sequences. These 13 were used for mitogenome characterization, comparative analyses, nucleotide composition bias, codon usage, and phylogenetic reconstruction. For the numbers of sequences used in other specific analyses (Table 2 and Table 3) and the corresponding exclusion criteria, see Section 2.6 in Methods.

**Table 2:** Analysis of length variation in the control region of *Aulacophora* mitogenomes.

Item	*A. indica* (N = 6)	*A. lewisii* (N = 6)
Total genome length (bp)		
Range	15,899–18,766	15,691–16,913
Mean ± SD	16,560 ± 1090	16,081 ± 442
Control region length (bp)		
Range	1299–4167	1113–2333
Mean ± SD	1963 ± 994	1502 ± 403
Gene overlap length (bp)		
Range	40–41	42–53
Mean ± SD	41 ± 0	46 ± 4
Intergenic region length (bp)		
Range	30–92	26–27
Mean ± SD	41 ± 25	27 ± 0

**Table 3:** Genetic diversity indices of the mitogenomes in *Aulacophora* species.

Species	Length (bp)	N	Haplotype Diversity (*Hd* ± SD)	Nucleotide Diversity (*Pi*)
*A. indica*	11,097	5	1.000 ± 0.126	0.00478
*A. lewisii*	11,097	5	1.000 ± 0.126	0.00198

### 2.2. Mitogenome Assembly and Annotation

In this study, next-generation sequencing (NGS) technology was employed to obtain mitogenome sequences. A genomic library with an insert size of 150 bp was constructed, and each sample was subjected to genome resequencing on the BGI platform. Raw sequence data were processed for quality control using fastp v0.20.0 (HaploX, Shenzhen, China) [26]. The mitogenomes was assembled de novo from the whole-genome sequencing reads using GetOrganelle v1.7.0 (Kunming Institute of Botany, CAS, Kunming, China) [27]. The locations of tRNA genes were predicted using the MITOS Web Server (University of Leipzig, Leipzig, Germany), with the invertebrate codon table selected [28]. For the 13 protein-coding genes (PCGs) and two ribosomal RNA (rRNA) genes, boundary determination was performed using SeqMan v7.1.0 (DNASTAR, Inc., Madison, WI, USA) software. The published mitogenome sequences of *A. indica* (NC_047467) [23] and *A. lewisii* (NC_039712) [21] were used as references for annotation.

### 2.3. Comparative Genomics Analysis

A mitogenome map was generated for visualization and comparison using Proksee (University of Alberta, Edmonton, AB, Canada) software [29]. MEGA v12 (Temple University, Philadelphia, PA, USA) software [30] was employed to calculate the base composition, codon usage bias, and strand skew characteristics of the mitogenome. The AT-skew and GC-skew were calculated according to the following formulas: AT-skew = (A − T)/(A + T); GC-skew = (G − C)/(G + C) [31].

### 2.4. Phylogenetic Analysis

Phylogenetic analysis included 13 individuals representing three *Aulacophora* species, with *Paragetocera flavipes* and *Agelocera mirabilis* designated as outgroups [20]. The 13 mitochondrial protein-coding genes were first translated into amino acids, aligned using MUSCLE in MEGA v12 (Temple University, Philadelphia, PA, USA) [30], and then back-translated to nucleotides. Ambiguously aligned regions were removed with Gblocks v0.91b (CSIC-UPF, Barcelona, Spain) [32] using default parameters (stringent mode: -b4 = 5, -b5 = h). The resulting filtered alignments were concatenated into a final supermatrix using BioEdit (Ibis Biosciences, Carlsbad, CA, USA) [33]. Phylogenetic relationships were inferred using IQ-TREE v1.6.12 (University of Vienna, Vienna, Austria) [34] under a codon model (-st CODON5), which partitions sequences by both gene and codon position (1st, 2nd, and 3rd), with independent substitution rates estimated for each partition. The best-fit substitution model for each partition was automatically selected based on the Bayesian Information Criterion (-m TEST -mrate G). Nodal support was assessed via 1000 ultrafast bootstrap replicates (-bb 1000).

### 2.5. Comparison of Genetic Diversity

To elucidate the population genetic structure and genetic diversity characteristics of the three *Aulacophora* species, we calculated the number of haplotypes, haplotype diversity (*Hd*), and nucleotide diversity (*Pi*) for each species using DnaSP v5.10.01 (University of Barcelona, Barcelona, Spain) software [35] based on the concatenated sequences of 13 protein-coding genes (PCGs) derived from the mitogenomes.

### 2.6. Explanation of Sample Size and Data Stratification Usage

This study newly sequenced the mitochondrial genomes of 11 individuals (5 *A. indica*, 5 *A. lewisii*, and 1 *A. nigripennis*). Together with two published GenBank sequences, the resulting 13-sequence dataset was used for genomic characterization, comparative analysis, nucleotide composition and codon usage, and phylogenetic reconstruction. The analysis of control region length variation (Table 2) was restricted to species represented by ≥5 individuals; *A. nigripennis* was therefore omitted, leaving 12 sequences. For genetic diversity and population structure analyses (Table 3), 10 sequences (5 *A. indica* and 5 *A. lewisii*) were retained, after excluding the two GenBank sequences, which lack precise locality data, and the single *A. nigripennis* sequence, for which population indices could not be computed. Discrepancies in sample sizes across tables and the corresponding exclusion criteria are explained in the text.

## 3. Results

### 3.1. General Features of Mitogenomes and Comparative Genomic Analysis

**Figure 1:**
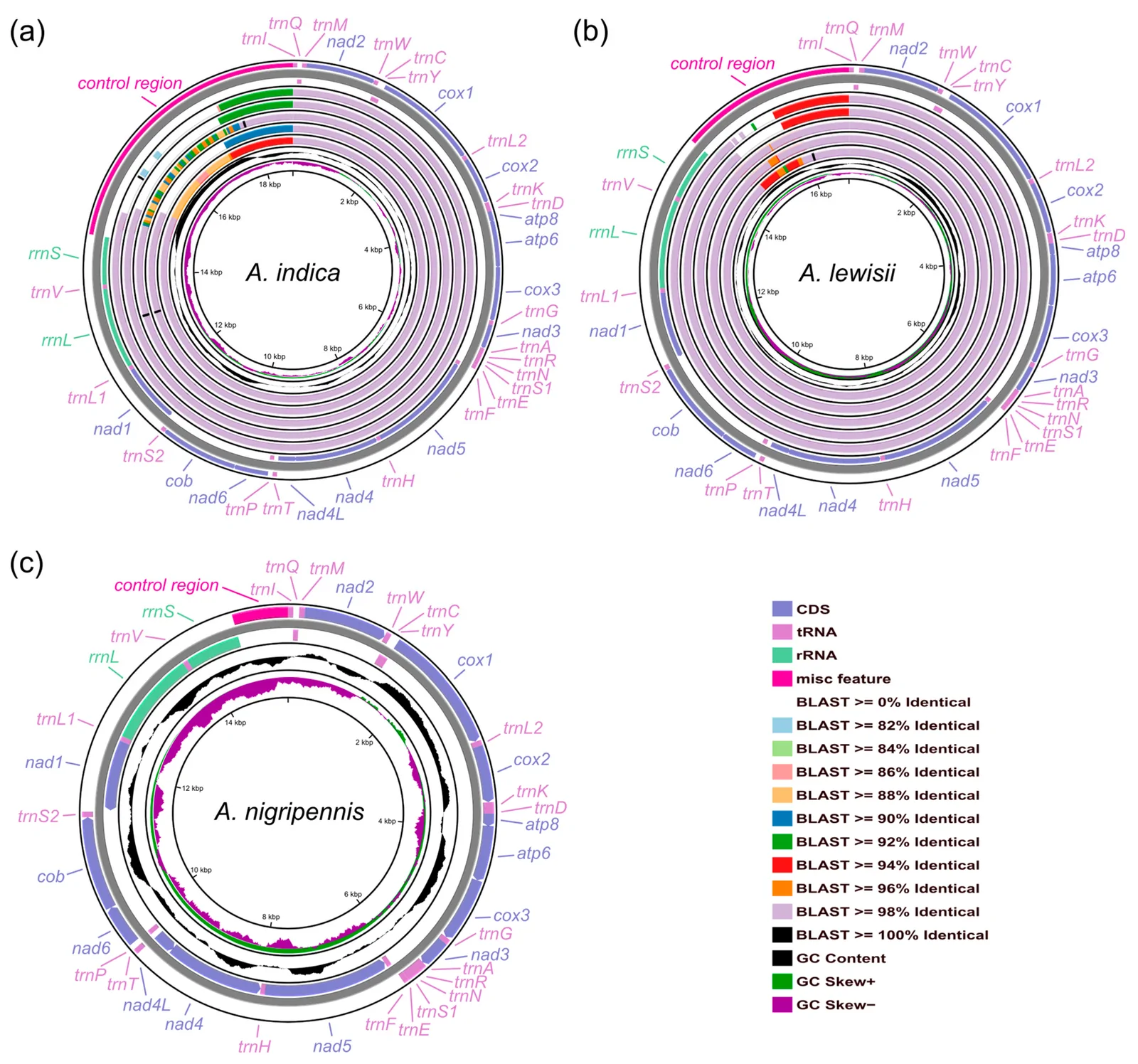
Circular maps of the complete mitogenomes of three *Aulacophora* leaf beetles. (**a**) *A. indica*; (**b**) *A. lewisii*; (**c**) *A. nigripennis*. The outer circle depicts the gene map, comprising 13 protein-coding genes (PCGs), 22 transfer RNA (tRNA) genes, two ribosomal RNA (rRNA) genes, and the AT-rich control region. tRNA genes are labeled with their corresponding one-letter IUPAC-IUB amino acid codes. Arrows indicate the direction of transcription. For *A. indica* and *A. lewisii*, gene colors reflect nucleotide identity based on intraspecific BLAST+ v2.14.1 (NCBI, Bethesda, MD, USA) comparisons among multiple individuals (N = 6 each), with the gradient ranging from red (high identity) to blue (low identity). For *A. nigripennis*, represented by a single individual, the color scheme indicates nucleotide identity relative to the consensus sequences of the other two species. The inner gray histogram shows GC content.

The complete mitogenome structures of three cucurbit leaf beetle species are presented in Figure 1 and Table 2. These genomes display the typical insect mitogenome composition, comprising a total of 37 genes: 13 protein-coding genes (PCGs), 22 transfer RNA (tRNA) genes, two ribosomal RNA (rRNA) genes, and a single non-coding control region (A + T-rich region). Comparative analysis revealed marked differences in total mitogenome length both within and among species. The mitogenome of *A. nigripennis*, for which only a single specimen was obtained, was 15,258 bp in length. Among species represented by multiple specimens, genome length ranged from 15,899 to 18,766 bp (N = 6) for *A. indica* and from 15,691 to 16,913 bp (N = 6) for *A. lewisii*. The genome structure of *A. lewisii* was the most compact and conserved, exhibiting the smallest average genome length (16,081 bp) and the lowest level of variation (SD = ±442 bp). Notably, although *A. indica* displayed the widest range in genome length, its average length (16,560 bp) was still greater than that of *A. lewisii*. Sequence identity analysis showed that PCGs and rRNA genes were highly conserved among species, with sequence identity exceeding 90%. In contrast, sequence identity within the control region was substantially lower, ranging from 65% to 78%. Further analysis revealed that length variation in the control region constituted the primary factor driving overall genome size differences. This variation was relatively stable in *A. lewisii* (range: 1113–2333 bp; mean: 1502 bp) and most polymorphic in *A. indica* (range: 1299–4167 bp; SD = ±994 bp). However, we note that the control region often contains repetitive sequences that are difficult to resolve accurately with short-read sequencing. In the absence of validation by long-read sequencing or PCR-based confirmation, the reported length ranges—particularly for highly variable regions—should be considered preliminary.

Furthermore, length variation in the intergenic regions (IGSs) exacerbated the differences in total genome length. This disparity was particularly pronounced in *A. indica*, which exhibited extensive IGS variation ranging from 30 to 92 bp with a standard deviation of 25 bp. In contrast, the IGS length in *A. lewisii* was highly stable, measuring 27 bp with a standard deviation close to zero. This finding further underscores the structural conservation of non-coding regions in this species.

### 3.2. Nucleotide Composition Bias and Codon Usage

**Figure 2:**
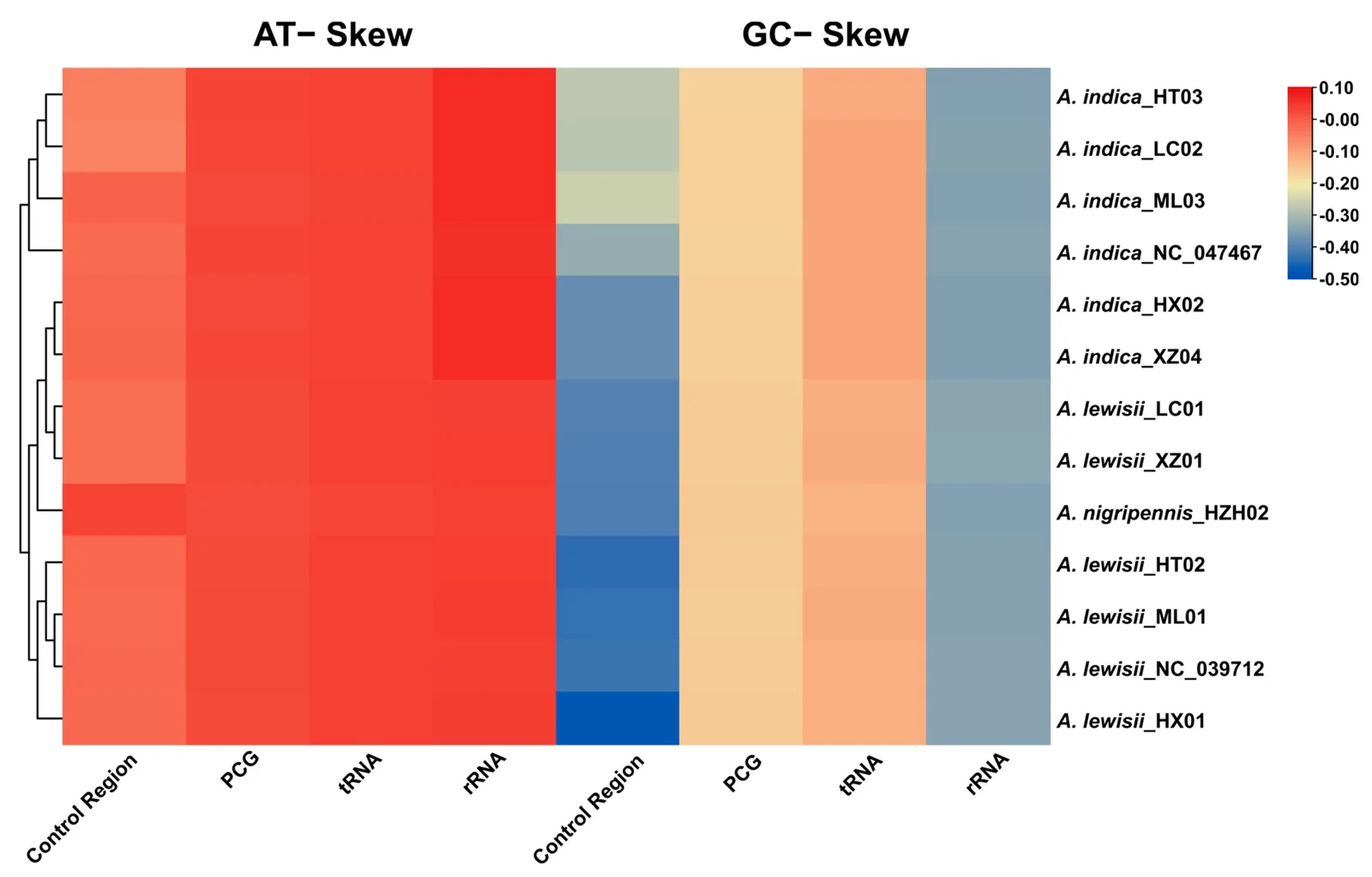
Nucleotide composition bias (AT-skew and GC-skew) of four genomic elements in the mitogenomes of three cucurbit leaf beetles. The *x*-axis and *y*-axis indicate the four genomic elements (Control Region, PCGs, tRNA, and rRNA) and the skew values, respectively.

The base composition and skew patterns of the mitogenomes of three cucurbit leaf beetle species are shown in Figure 2. All examined species displayed a marked AT bias across their mitogenomes, reflected in consistently positive AT−skew values and negative GC−skew values overall. Within protein-coding genes (PCGs), AT-skew values for *A. indica*, *A. lewisii*, and *A. nigripennis* ranged from 0.024 to 0.031, while GC−skew values ranged from −0.169 to −0.155, indicating a general bias toward adenine over thymine and cytosine over guanine in coding sequences. Transfer RNA (tRNA) and ribosomal RNA (rRNA) genes both maintained positive AT−skew values, ranging from 0.030 to 0.038 and from 0.039 to 0.067, respectively, whereas their GC−skew values were uniformly negative, from −0.120 to −0.102 in tRNAs and from −0.348 to −0.335 in rRNAs. The rRNA regions exhibited substantially more negative GC−skew values than the tRNA regions, pointing to a pronounced depletion of guanine in this genomic compartment. In contrast, the control region showed the greatest variability: AT−skew values ranged widely from −0.055 to 0.034, and GC−skew values fluctuated considerably, from −0.478 to −0.257, revealing pronounced interspecific divergence in nucleotide composition. Overall, base composition biases were largely conserved across most mitochondrial compartments of the three species, with the control region and rRNA genes accounting for the primary interspecific variation.

**Figure 3:**
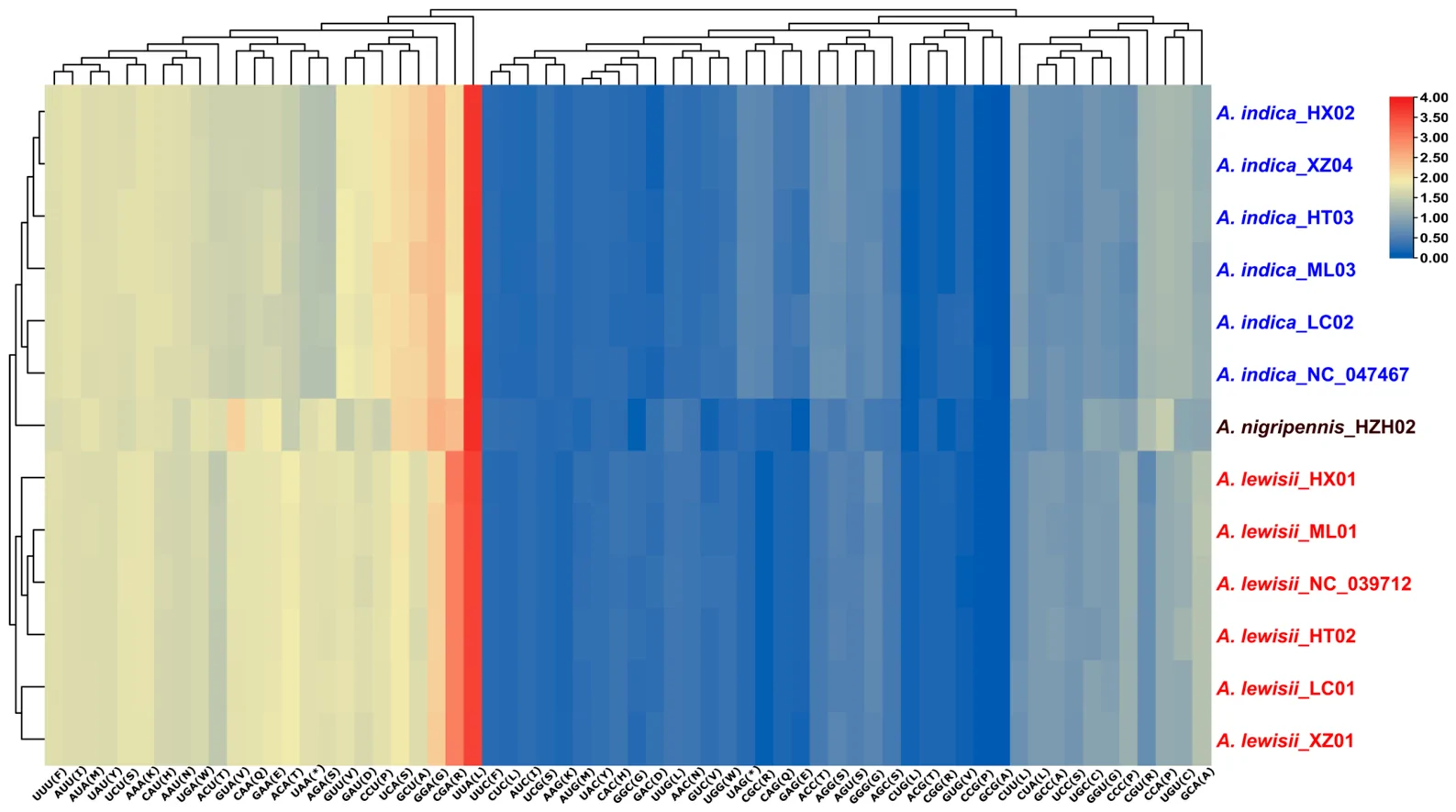
The relative synonymous codon usage (RSCU) of PCGs in three cucurbit leaf beetles. The *x*-axis and *y*-axis indicate the hierarchical clustering of codon frequencies and three cucurbit leaf beetle species, respectively. * represents the stop codon.

Relative synonymous codon usage (RSCU) analysis revealed marked interspecific divergence in codon usage among the three *Aulacophora* species, while intraspecific patterns remained highly consistent (Figure 3). A pronounced bias toward UUA (Leu) was universally observed, with RSCU values ranging from 3.59 to 3.82, representing the highest peak in the heatmap. In contrast, codons such as CCG (Pro) and GCG (Ala) were rarely utilized, with RSCU values approaching zero (0.03–0.09 and 0.00–0.05, respectively), and CGC (Arg) also exhibited extremely low usage (0.10–0.61). Interspecific comparisons uncovered codon preferences that paralleled phylogenetic relationships. *A. lewisii* showed a significantly elevated preference for CGA (Arg) (RSCU 3.00–3.08), markedly higher than that in *A. indica* (1.94–2.06) and *A. nigripennis* (2.40). Additionally, *A. lewisii* displayed relatively high usage of CAA (Gln) and CAG (Gln) (consistently above 1.80 and 0.20, respectively), whereas these codons were generally less frequent in the other two species. Notably, *A. nigripennis*, phylogenetically closer to *A. indica*, exhibited a unique preference for GUA (Val) (RSCU 2.16) and a strikingly low usage of GAG (Glu) (RSCU 0.07), both deviating from the patterns observed in the other species. These distinct biases suggest that the three species may have experienced different evolutionary selection pressures at the synonymous codon level.

### 3.3. Genetic Diversity and Population Structure

This study used haplotype diversity (*Hd*) and nucleotide diversity *(Pi*) to assess genetic diversity and differentiation among *Aulacophora* populations. Higher *Hd* and *Pi* values denote greater genetic variation within a population. In mitochondrial DNA, elevated diversity can reflect historically large effective population sizes or stable demographic histories, potentially supplying the raw material for adaptive responses—though functional inference requires corroborating nuclear or genomic evidence [36,37]. Because sample availability was limited for several species, genetic diversity was assessed only in *A. indica* and *A. lewisii*. Both species exhibited the maximum haplotype diversity (*Hd* = 1.000). However, nucleotide diversity in *A. indica* (*Pi* = 0.00478) was approximately 2.4-fold higher than in *A. lewisii* (*Pi* = 0.00198), indicating substantially greater intraspecific genetic variation in *A. indica*.

### 3.4. Phylogenetic Relationships

**Figure 4:**
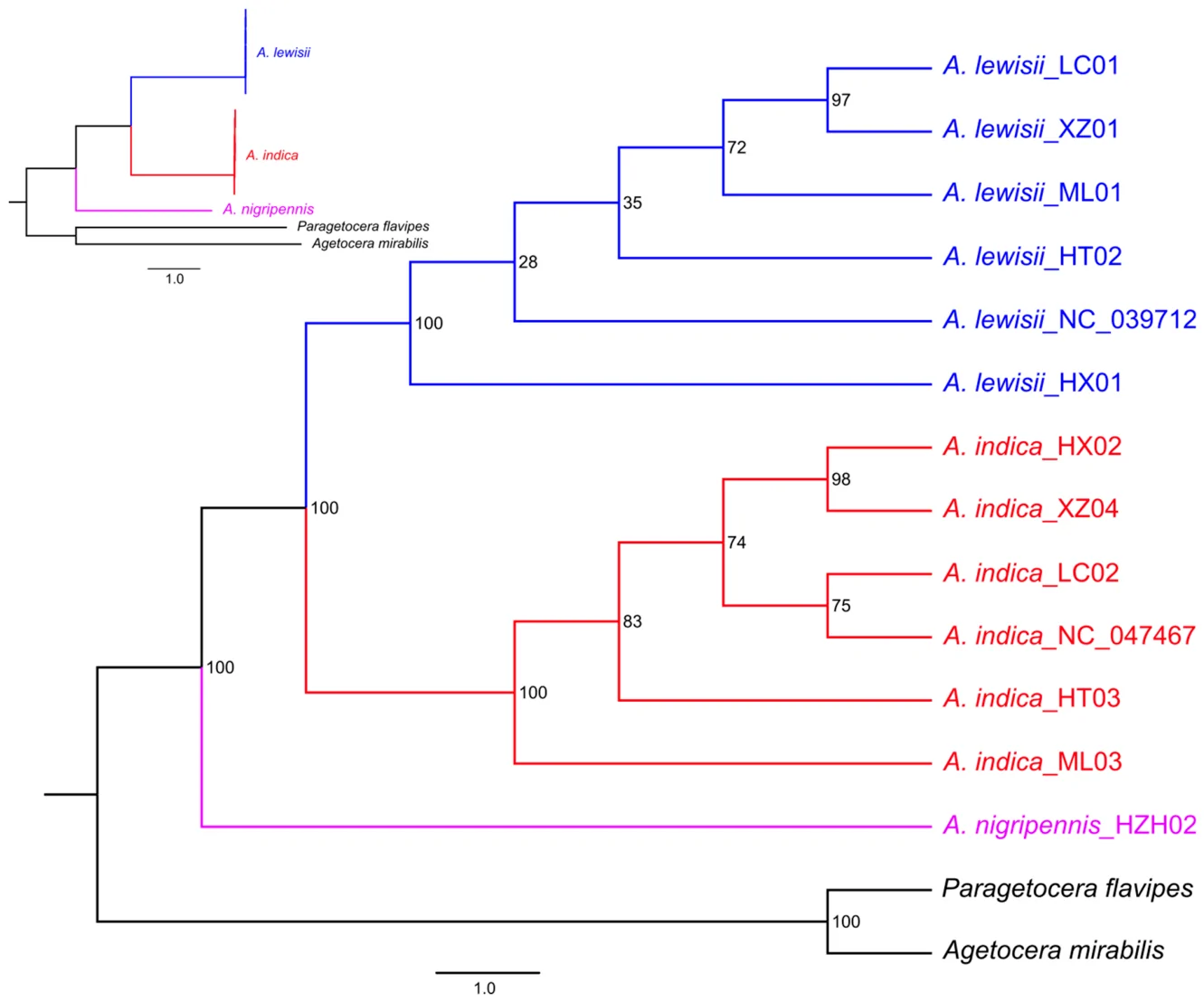
Phylogenetic tree inferred from mitogenomes of three *Aulacophora* species. Numbers at the nodes are ML bootstrap values.

Phylogenetic relationships among *Aulacophora* species were inferred from 13 mitochondrial PCGs, with *Paragetocera flavipes* and *Agelocera mirabilis* as outgroups (Figure 4). The resulting topology resolved *A. nigripennis* as the basal lineage. Among the remaining taxa, *A. lewisii* and *A. indica* were recovered as closely related sister species. All major interspecific nodes received 100% bootstrap support. The monophyly of *A. lewisii* and *A. indica* was strongly supported. However, bootstrap values for intraspecific relationships within these species were generally low (<80%), indicating weak resolution of terminal branching patterns.

## 4. Discussion

This study presents the first systematic comparative analysis of the mitogenomes of three *Aulacophora* pest species. The analysis elucidates similarities and differences in their genome organization, base composition, codon usage bias, phylogenetic relationships, and population genetic diversity. These findings collectively provide an important molecular basis for understanding the evolutionary background, ecological adaptation, and genetic differentiation within this genus.

Our results showed that the mitogenomes of all three species contained the typical set of 37 genes. Their gene order was highly consistent, and no gene rearrangement events were detected. This finding aligns with the conserved genomic architecture reported in Coleoptera and most other insects [1,2,38]. Such structural conservation reflects strong functional constraints acting on the mitogenome during evolution [2]. Despite this conserved gene order, total mitogenome length varied markedly among the three *Aulacophora* species (15,258–18,766 bp) (Figure 1). These differences were primarily attributable to length variation in the control region, which contributed approximately 80% of the total variation, while intergenic regions and gene overlaps accounted for only minor contributions (Table 2). As a non-coding region under relatively weak functional constraint, the control region is prone to insertions, deletions (indels), and the expansion or contraction of tandem repeats [39,40]. Given that short-read sequencing has inherent limitations in resolving complex repetitive regions, the exact boundaries, repeat structures, and absolute lengths of the control region reported here may be subject to uncertainty. Accordingly, conclusions regarding the causes of mitogenome length variation should be regarded as tentative pending further validation. Consequently, it represents the primary source of length variation in mitogenomes [41,42,43]. Notably, the pattern of control region length variation differed significantly among species. *A. lewisii* exhibited the most stable control region length (1113–2333 bp; mean 1502 ± 403 bp). In contrast, *A. indica* displayed considerable length fluctuations, with a range of 1299–4167 bp and a high standard deviation of ±994 bp. This species also exhibited the most prominent length variation in intergenic regions, further underscoring the high genomic plasticity inherent to its non-coding regions. Whether these interspecific differences in mitogenome length variation are related to host range, population structure, or other ecological factors remains an open question for future investigation.

The mitogenomes of the three *Aulacophora* species all exhibited a pronounced AT bias, a feature consistent with most insect mitogenomes [2,23]. Within the protein-coding genes (PCGs), positive AT−skew and negative GC-skew values were observed, indicating strand asymmetry in nucleotide composition. Relative synonymous codon usage (RSCU) analysis further revealed significant interspecific variation in codon usage. Notably, *A. lewisii* displayed a markedly higher preference for the CGA (R) codon relative to the other species, whereas *A. nigripennis* exhibited a distinct usage profile across multiple codons. These observed differences may reflect varying evolutionary constraints, including selection pressure, mutational bias, or translational efficiency [44,45].

Phylogenetic analysis based on the 13 protein-coding genes revealed that the strongly supported basal position of *A. nigripennis* distinguishes it as an early-diverging lineage within the genus and reveals a deep evolutionary split between this species and the *A. lewisii*–*A. indica* clade. The latter pair was recovered with maximum nodal support, consistent with prior phylogenetic hypotheses and reinforcing their close relationship. Despite the robust resolution at interspecific nodes, the consistently low bootstrap support within each of these two species (generally <80%) indicates that the 13 mitochondrial PCG dataset provides limited power for resolving shallow intraspecific divergences. This pattern is likely attributable to recent or rapid diversification events within these lineages, coupled with the known constraints of a single-locus mitochondrial marker in capturing fine-scale population-level splits.

The population genetic analysis revealed that *A. indica* exhibited the highest nucleotide diversity (*Pi* = 0.00478), followed by *A. lewisii* (*Pi* = 0.00198). Phylogenetically, *A. lewisii* and *A. indica* were recovered as closely related sister species, and neither represents an early-diverging lineage within the genus. Although *A. lewisii* is an oligophagous species that feeds on a limited range of host plants, such as *Luffa* spp. [12,46], its haplotype diversity was notably high (*Hd* = 1.000). *A. indica*—a polyphagous species with a broader host range—also displayed the maximum haplotype diversity *(Hd* = 1.000) but with approximately 2.4-fold higher nucleotide diversity. The markedly higher nucleotide diversity observed in *A. indica* may point to a greater reservoir of standing genetic variation, a pattern that could reflect differences in demographic history between the two species. However, in the absence of direct demographic analyses (e.g., Tajima’s *D*, mismatch distribution) and corroborating nuclear genomic evidence, specific historical scenarios—such as population expansion versus long-term stability—remain speculative. This contrast in nucleotide diversity, set against a shared background of high haplotype diversity, suggests that host-plant specialization and population history may have differentially shaped the genetic architectures of these two species.

This study systematically elucidated, for the first time, the genetic characteristics and evolutionary relationships of three *Aulacophora* pest species based on mitogenome data. Nonetheless, several limitations should be acknowledged. First, sample representativeness was limited, as evidenced by the inclusion of only a single specimen of *A. nigripennis*, which constrains a comprehensive assessment of its intraspecific genetic diversity and population structure. Second, the scope of this investigation was confined to the mitogenome and did not incorporate nuclear gene variation, the latter often being more intimately linked to adaptive evolution. Future investigations should therefore expand geographic and taxonomic sampling, integrating whole-genome sequencing, demographic history modeling, and functional gene validation. Such integrated approaches are imperative to decipher the causal interplay among dietary breadth, host selection, and genetic diversity, ultimately providing a more robust theoretical scaffold underpinning the evolutionary trajectories and integrated pest management of *Aulacophora* species.

## 5. Conclusions

This study presents a comparative analysis of the mitochondrial genomes of three *Aulacophora* pests (*A. indica*, *A. lewisii*, and *A. nigripennis*). The three mitogenomes showed conserved gene order but differed substantially in total length, driven primarily by variation in the control region; all species displayed pronounced AT bias, and interspecific differences in codon usage were observed. Phylogenetic analysis based on 13 protein-coding genes supported *A. nigripennis* as the basal lineage and *A. lewisii* and *A. indica* as sister species. Both widespread species exhibited high haplotype diversity, yet *A. indica* showed approximately 2.4-fold higher nucleotide diversity than *A. lewisii*, indicating differential population genetic architectures between the two species. Although constrained by limited sample sizes, taxonomic coverage, and preliminary control region lengths, these findings provide baseline data for comparative mitogenomics of *Aulacophora* and lay a foundation for future phylogenetic and population genetic studies with broader sampling.

## Data Availability

Data to support this study are available from the National Center for Biotechnology Information (https://www.ncbi.nlm.nih.gov) (accessed on 20 December 2025). The GenBank numbers are PZ334452–PZ334461 and PZ358530–PZ358535.
